# CD73low B-cell phenotypes and distinct cytokine profiles in patients with active anti-Jo-1 antibody positive idiopathic inflammatory myopathies

**DOI:** 10.1136/rmdopen-2024-005401

**Published:** 2025-04-09

**Authors:** Maho Nakazawa, Begum Horuluoglu, Charlotte de Vries, Karin Lodin, Vivianne Malmström, Ingrid E Lundberg, Caroline Grönwall

**Affiliations:** 1 Division of Rheumatology, Department of Medicine, Solna, Karolinska Institutet, Stockholm, Karolinska University Hospital, Center for Molecular Medicine, Stockholm, Sweden; 2 Japan Society for the Promotion of Science, Tokyo, Japan; 3 Division of Rheumatology, Department of Internal Medicine, Keio University School of Medicine, Tokyo, Japan; 4 Department of Gastro, Dermatology and Rheumatology, Karolinska University Hospital, Stockholm, Sweden

**Keywords:** B cells, Dermatomyositis, Polymyositis

## Abstract

**Objectives:**

We performed multiparameter phenotyping of peripheral B cells in anti-Jo-1 antibody positive idiopathic inflammatory myopathies (IIM) to delineate disease-associated immunological profiles and the influence of B cells on disease activity.

**Methods:**

Purified B cells from peripheral blood mononuclear cells from 16 patients with anti-Jo-1 antibody positive IIM (7 with untreated active IIM, 4 with active and treated IIM and 5 with inactive IIM) were analysed by multiparameter spectral flow cytometry. Dimensionality reduction and clustering analysis were applied to pre-gated CD19+B cells. Serum levels of 21 cytokines and anti-Jo-1 IgG autoantibodies were determined. All patients with IIM in this study were positive for anti-Jo-1 antibody.

**Results:**

Anti-Jo-1 antibody levels correlated positively to disease activity. Flow cytometry demonstrated B-cell dysregulation with significantly lower CD73 expression on naïve, switched memory and double negative B cells in patients with active IIM. Clustering analysis further revealed expansions of CD73− IgM+naïve B cells and CD73− CD95+ switched memory B cells in active IIM. In unswitched memory B cells, CD73+CD21+ cells were decreased in active IIM. Patients with active IIM had significantly higher serum levels of B-cell activating factor, inducible protein-10, interleukin-6 and sCD40L which correlated with changes in B-cell populations.

**Conclusions:**

Since CD73 has an immunoregulatory function by modulating the ATP/adenosine pathway, which is also targeted by methotrexate, the low CD73 B-cell expression in anti-Jo-1 antibody-positive IIM may lead to B-cell hyperactivation. These novel findings further highlight B cells as central in the pathogenesis of IIM and important therapeutic targets.

**WHAT IS ALREADY KNOWN ON THIS TOPIC**

- B cells have important roles in the pathogenesis of anti-Jo-1 antibody positive idiopathic inflammatory myopathies (IIM) which have a poor prognosis with risk of lung involvement.

**WHAT THIS STUDY ADDS**

- This well-characterised IIM cohort showed distinct B-cell phenotypes and cytokine profiles in active anti-Jo-1 antibody positive IIM.
- CD73− B cell subpopulations and activated B-cell subpopulations were expanded in anti-Jo-1 antibody positive IIM and correlated with disease activity, levels of proinflammatory cytokines and anti-Jo-1 antibodies.
- This study indicated a link between the skewed CD73− B-cell subpopulations, the ATP/adenosine pathway and the inflammatory cytokine profiles in anti-Jo-1 antibody positive IIM.

**HOW THIS STUDY MIGHT AFFECT RESEARCH, PRACTICE OR POLICY**

- Low CD73 expression on B cells may lead to hyperactivation of B cells through the ATP/adenosine pathway and CD73 restoration can be a treatment target in anti-Jo-1 antibody positive IIM.

## Introduction

 Idiopathic inflammatory myopathies (IIM) or myositis are autoimmune disorders characterised by inflammation of skeletal muscles, but also affect extramuscular organs such as skin, joints, heart and lungs.^1^ Interstitial lung disease (ILD) is a common manifestation occurring in 20–40% of IIM and is a major cause of IIM mortality.^2^,^4^ Even though IIM is a recurrent, refractory and sometimes fatal disease, the pathological mechanisms of IIM are not fully revealed.

Autoantibodies are present in up to 80% of the patients with IIM indicating a role of B cells in the pathogenesis of this disorder.^5^ Anti-Jo-1 IgG is the most common autoantibody type, found in 20–30% of IIM.^6 7^ Importantly, the presence of anti-Jo-1 antibodies is strongly associated with ILD, which is seen in up to 90% of anti-Jo-1 positive patients.^8^ Treatment of patients with IIM includes glucocorticoids and immunosuppressive agents which can act broadly on various kinds of immune cells. B-cell targeting anti-CD20 therapy with rituximab has been reported to be more effective in anti-Jo-1 antibody positive disease than other subgroups of IIM,^9^,^12^ supporting a role of B cells in this subset of IIM. This is further emphasised by case reports showing a striking efficacy of CD19-targeted chimeric antigen receptor T cell therapy in refractory anti-Jo-1 antibody positive IIM.^13 14^ However, this heavy immune suppression may be associated with side effects and will most likely be limited to patients with life-threatening disease. Therefore, it is attractive to identify more specific target molecules on pathogenic B cells.

Previous investigations of IIM have shown that in peripheral blood, the frequencies of naive B cells could be increased while memory B cells decreased compared with healthy controls.^15^,^19^ Circulating plasmablast frequencies have been suggested to be elevated in IIM compared with controls based on a recent report.^19^ However, this may vary depending on autoantibody-positive subsets, as another study demonstrated the high frequency of plasmablasts, particularly in anti-melanoma differentiation associated gene 5 antibody positive patients compared with other IIM subsets.^20^ Importantly, it is still unclear which subpopulations of B cells are important in the anti-Jo-1 antibody positive subset of IIM. Since the proportions of B-cell subpopulations vary with disease activity or autoantibody profiles in IIMs,^20^ studies in clinically and serologically well-characterised cohorts are essential.

Several B-cell surface markers can be used to identify distinct B-cell subsets and B-cell activation states in association with autoimmunity. For instance, CD95+memory B-cell subset and CD11c+memory B-cell subsets have both been found to be expanded in autoimmune diseases. CD95 is expressed on activated B cells,^21 22^ and CD95+memory B cells have high sensitivity to immune stimulation.^23^ CD11c is considered a marker of atypical memory B cells which express low or no density of CD27 and CD21 and may reflect a state of activation by chronic stimulation.^24^ A less-studied pair of surface markers expressed by B cells are CD39 and CD73, both of which are enzymes related to the ATP/adenosine pathway. They have immunoregulatory functions and play an important role for immunometabolism due to their adenosine suppressive functions;^25^,^27^ however, they are not well characterised in the context of B cells in autoimmune diseases.

To further advance B-cell targeted treatment, more in-depth knowledge about B-cell subsets in anti-Jo-1 antibody positive IIM is necessary. In this study, we performed multiparameter spectral flow cytometry B-cell phenotyping of well-characterised patients with anti-Jo-1 antibody positive IIM in different phases of disease, inflammatory active with and without immunosuppressive treatment and inactive disease. The immunological profiles revealed B-cell distortion in patients with active IIM and in particular a low CD73 B-cell expression.

## Methods

### Patients with IIM

16 patients with anti-Jo-1 positive IIM attending the Rheumatology clinic and Respiratory Medicine Unit at Karolinska University Hospital (Stockholm, Sweden) were enrolled. All patients in this study were diagnosed with definite or probable IIM classified by 2017 European Alliance of Associations for Rheumatology (EULAR)/American College of Rheumatology (ACR) classification criteria for IIM^28^ and were positive for anti-Jo-1 antibody at their first visit to the clinic. Anti-Jo-1 antibody positive IIM could also be called anti-Jo-1 antibody positive antisynthetase syndrome. However, we used the EULAR/ACR classification for IIM to define our cohort, since the new classification criteria for antisynthetase syndrome have not yet been published. The clinical data were collected from medical charts. Age-matched and sex-matched samples (8 for flow cytometry and 17 for serum studies) from healthy individuals were obtained from Karolinska University Hospital and Karolinska Institutet. Disease activity was evaluated based on the juvenile dermatomyositis criteria.^29^ The definition of ‘inactive’ disease in this study was that patient global assessment was less than 2 cm of 10 cm on a Visual Analogue Scale and at least two of the following three criteria were fulfilled; manual muscle test eight score was >78 of 80, health assessment questionnaire score was <0.5 of 3 or the level of creatine kinase (CK) was <2.5 ukat/L. The definition of ‘active’ disease was when the definition of ‘inactive’ was not fulfilled. According to this definition of ‘active’ and ‘inactive’ disease activity, the patients were classified into three groups: untreated active IIM group, treated active IIM group and inactive IIM group. Written informed consent was obtained from all patients and the healthy donors in this study. This study was conducted in accordance with the Declaration of Helsinki.

### Blood samples for immunophenotyping

Peripheral blood mononuclear cells (PBMC) were isolated by a Ficoll-Paque PLUS gradient (Cytiva, Uppsala, Sweden) and cryopreserved in DMSO (dimethyl sulfoxide) and stored in liquid nitrogen until used. After thawing, CD19+B cells were negatively purified by using the EasySep Human B Cell Isolation Kit (STEMCELL Technologies, Vancouver, Canada). The B cells were stained with a 23-colour panel for 30 min at 4° and washed with staining buffer (phosphate-buffered saline with 0.5% BSA (bovine serum albumin) and 2 mM EDTA) and data was acquired by a four-laser Cytek Aurora spectral cytometer (Cytek, Fremont, USA, online supplemental table 1).

### Clustering analysis

CD19+B cells were gated and down-sampled to 1500 counts per sample from the flow cytometry data and concatenated. Dimensionality reduction analysis on the merged B cells data was visualised by Uniform Manifold Approximation and Projection (UMAP) and flow self-organising maps (FlowSOM) analysis was performed by FlowJo software V.10.10 (TreeStar, Ashland, USA).

### Serum analyses for anti-Jo-1 antibody and cytokines

A total of 21 cytokines were measured in serum samples from the 16 patients with IIM and 17 control samples by using a 13-plex LEGENDplex Human B Cell Panel which detected tumour necrosis factor (TNF)-α, interleukin (IL)-13, IL-4, IL-10, IL-6, IL-2, TNF-β, interferon (IFN)-γ, IL-17A, IL-12p70, a proliferation-inducing ligand (APRIL), B-cell activating factor (BAFF) and soluble CD40 ligand (sCD40L) and a customised panel including IL-1β, IFN-γ inducible protein (IP)-10, IFN-α2, IFN-β, IFN-λ1, IFN-λ2/3, IL-8, granulocyte-macrophage colony-stimulating factor (BioLegend, San Diego, USA) according to the manufacturer’s instructions. The cytokines were quantified using a four-laser Cytek Aurora spectral cytometer (Cytek, Fremont, California, USA). The levels of anti Jo-1 IgG antibodies were measured in serum by an in-house ELISA as previously described.^30^

### Statistical analysis

Statistical comparisons were performed using the Mann-Whitney U test between two groups and the Kruskal-Wallis test between three groups. Correlations of variables were analysed using Spearman’s test and the best fit lines were shown. The results were considered significant at a value of p<0.05. All statistical analyses were conducted with JMP V.17.0 software (SAS Institute, North Carolina, USA).

## Results

### In-depth flow cytometry analysis of anti-Jo-1 antibody patients with positive IIM

**Figure 1:**
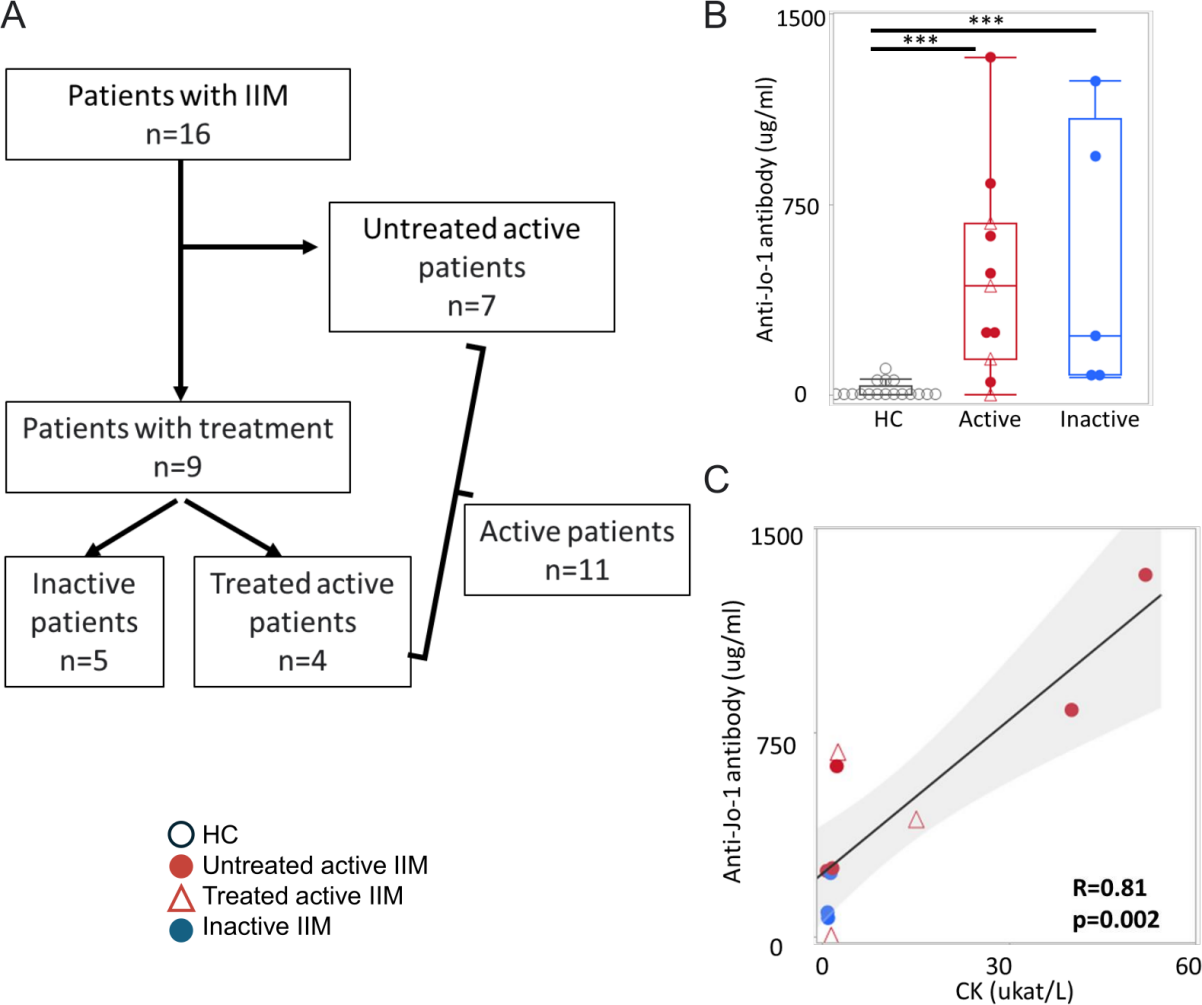
Overview of the investigated anti-Jo-1 antibody positive patients. (**A**) Schematic overview of the included patients for flow cytometry analysis. (**B**) The serum levels of anti-Jo-1 antibody in active IIM (n=11), inactive IIM (n=5) and healthy controls (HC, n=17) by ELISA. The median±IQR is shown. ***p<0.001 by Mann-Whitney U test. (**C**) Spearman’s correlation analysis between anti-Jo-1 antibody levels and CK levels. The HC, untreated active, treated active and inactive IIM groups are indicated by black circles, red circles, red triangles and blue circles, respectively. CK, creatine kinase; IIM, idiopathic inflammatory myopathies.

The peripheral B-cell phenotype of 16 well-characterised patients with anti-Jo-1 antibody positive IIM and 8 age-matched and sex-matched controls was analysed using a 23-marker spectral flow cytometry panel on purified blood B cells (figure 1A, table 1). Among the nine treated patients, five patients were clinically stable and the other four patients had active disease according to the definition for disease activity. The untreated active group (n=7) and the treated active group (n=4) were grouped together as active IIM (n=11). All patients except one treated patient were confirmed to be IgG anti-Jo-1 positive at the time of blood sampling by in-house ELISA, and all healthy controls were negative. The one patient who was negative by in-house ELISA had been anti-Jo-1 antibody positive at diagnosis before treatment. The anti-Jo-1 antibody levels among the patients with active IIM displayed a significant positive correlation with serum levels of CK, which is an indicator of active muscle inflammation (figure 1B,C).

**Table 1:** Characteristics of the patients and healthy controls

	HC for flow cytometry cohort	HC for serum cohort	Inactive IIM	Active IIM		
				Untreated active IIM	Treated active IIM	Total
	n=8	n=17	n=5	n=7	n=4	n=11
Mean age (years)	47.6±17.6	51.9±11.3	52.0±15.1	50.7±15.8	55.5±7.8	52.5±13.2
Female, n (%)	6 (75)	11 (64.7)	4 (80)	4 (57.1)	2 (50)	6 (54.6)
Anti-Jo-1 antibody positivity, n (%)	N/A	N/A	5 (100)	7 (100)	7 (100)	11 (100)
Anti-Ro52 antibody positivity, n (%)†	N/A	N/A	5 (100)	4 (57.1)	1 (25)	5 (45.5)
Median duration from disease onset to sampling (months)	N/A	N/A	11 (3.5–42)	5 (2–21)	74.5 (16.3–156.8)	6 (2–84)
Treatment						
Untreated, n (%)	N/A	N/A	0 (0)	7 (100)	0 (0)	7 (63.6)
PSL, n (%)	N/A	N/A	5 (100)	N/A	4 (100)	4 (36.4)
Median dose of PSL (mg/day)	N/A	N/A	10 (5.6–16.3)^*^	N/A	7.5 (5–25)	0 (0–5)
MTX, n (%)	N/A	N/A	1 (20)	N/A	2 (50)	2 (18.2)
AZA, n (%)	N/A	N/A	1 (20)	N/A	1 (25)	1 (9.1)
MMF, n (%)	N/A	N/A	2 (40)	N/A	0 (0)	0 (0)
Disease activity						
Mean MMT-8 score	N/A	N/A	79.4±0.9	74.1±9.5	69±4.2	72.3±8.1
Median physician’s global assessment (VAS)	N/A	N/A	0.5 (0–1.9)‡	5.0 (2.0–5.4) n=5	2.5 (2.0–4.5) n=3	3.5 (2.0–5.0) n=8
Median patient’s global assessment (VAS)	N/A	N/A	0.8 (0.2–1.0)‡	4.3 (3.8–5.4) n=6	5.3 (4.7–5.6)	4.8 (4.1–5.5) n=10
Median HAQ total score	N/A	N/A	0 (0–0.3)‡	1.0 (1.0–1.4)	1.2 (1.0–1.8)	1.0 (0.8–1.6) n=9
Median extramuscular disease-activity score	N/A	N/A	5 (0–14.5)‡	2.5 (1.6–4.4) n=4	2.0 (2.0–2.4) n=3	2.0 (1.9–3.0) n=7
Median creatine kinase (ukat/L)	N/A	N/A	0.9 (0.8–1.3) n=3	2.3 (1.2–46.1) n=5	2.5 (1.4–15.1) n=3	2.4 (1.5–33.9) n=8

Mean and median are shown as mean±SD and median (first quartile - —third quartile), respectively.

*p<0.05, significantly higher than active IIM by Mann-Whitney U test.

†Only anti-Ro52 antibody was found among myositis-associated antibodies.

‡p<0.001, significantly lower than active IIM by Mann-Whitney U test.

AZA, azathioprine; HAQ, Health Assessment Questionnaire; HC, healthy controls; IIM, idiopathic inflammatory myopathies; MMF, mycophenolate mofetil; MMT-8, Manual Muscle Test–8; MTX, methotorexate; PSL, prednisolone; VAS, Visual Analogue Scale.

### Distortions were observed in B-cell populations in patients with anti-Jo-1 antibodies

**Figure 2:**
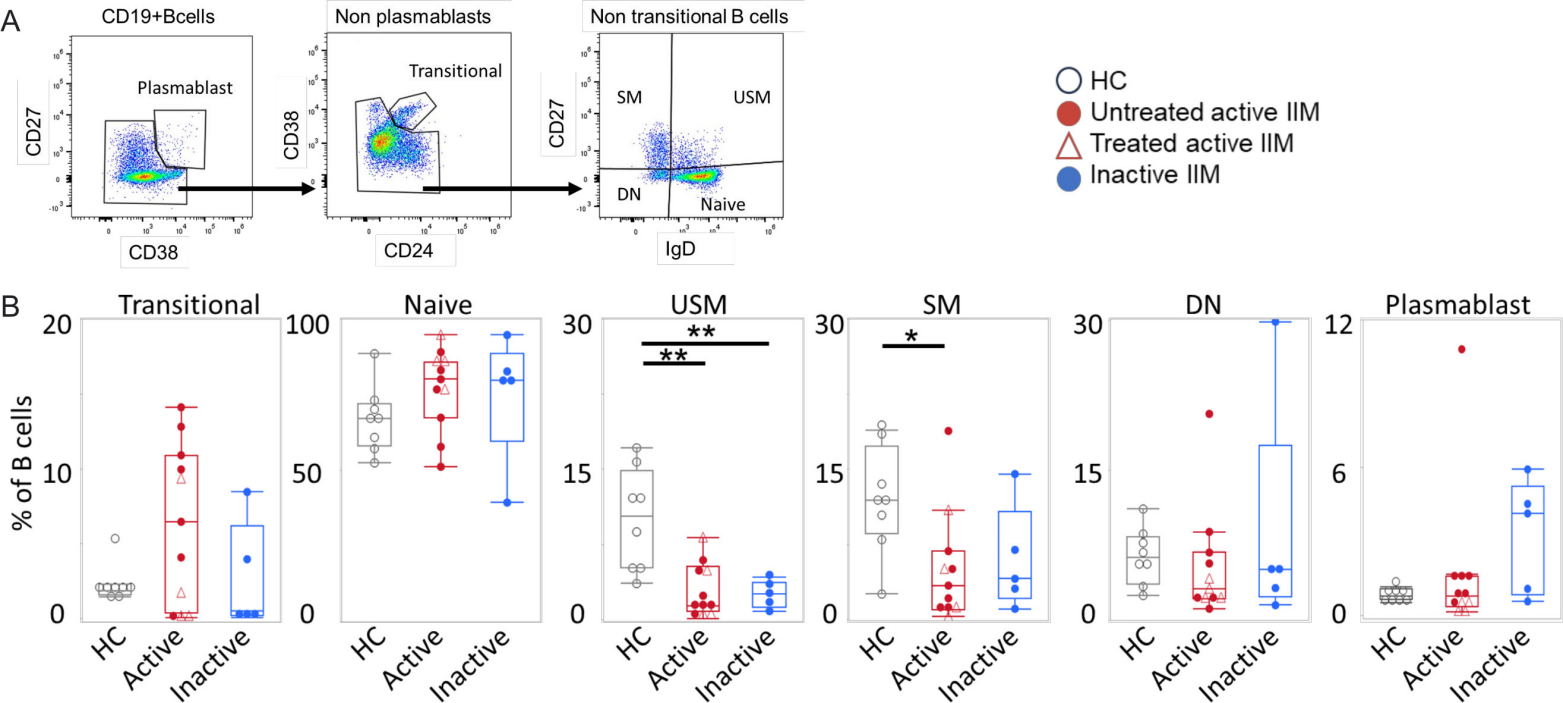
Comparison of major B-cell subpopulations. Results from flow cytometry B cell phenotyping in IIM. (**A**) Representative gating of plasmablasts, transitional B cells, naïve B cells, unswitched memory (USM) B cells, switched (SM) memory B cells and CD27− IgD− double negative (DN) B cells. (**B**) Percentages of transitional, naïve, USM, SM, DN B cells and plasmablasts among CD19+B cells. The active IIM (n=11), inactive IIM (n=5) and HC (n=8) groups are coloured red, blue and black, respectively. The median±IQR is shown. *p<0.05, **p<0.01 by Mann-Whitney U test. HC, healthy controls; IIM, idiopathic inflammatory myopathies.

First, we analysed the flow cytometry data with conventional manual gating and compared the classical subpopulations of peripheral B cells from patients with active IIM (n=11), inactive IIM (n=6) and healthy controls (HC, n=8; figure 2). The frequency of B cells in PBMCs was not different among the three groups (online supplemental table 2). The gating strategy is shown in figure 2A identifying: plasmablasts as CD19+CD27+CD38+; transitional B cells as CD19+ CD24^hi^ CD38+B cells among whole B cells minus plasmablasts; naïve B cells, unswitched memory (USM) B cells, switched memory (SM) B cells and double-negative (DN) B cells were respectively IgD+CD27−, IgD+CD27+, IgD− CD27+ and IgD− CD27− CD19+B cells among whole B cells minus the subpopulations of plasmablasts and transitional B cells. The majority of B cells were CD27− IgD+naïve B cells in all groups, but there were no significant differences between the three groups (figure 2B). The memory B cells were significantly lower in the active IIM group than in HC and transitional B cells were increased in patients with untreated active compared with controls (figure 2B, online supplemental figure 1).

### Low CD73 expression on B cells in active IIM

**Figure 3:**
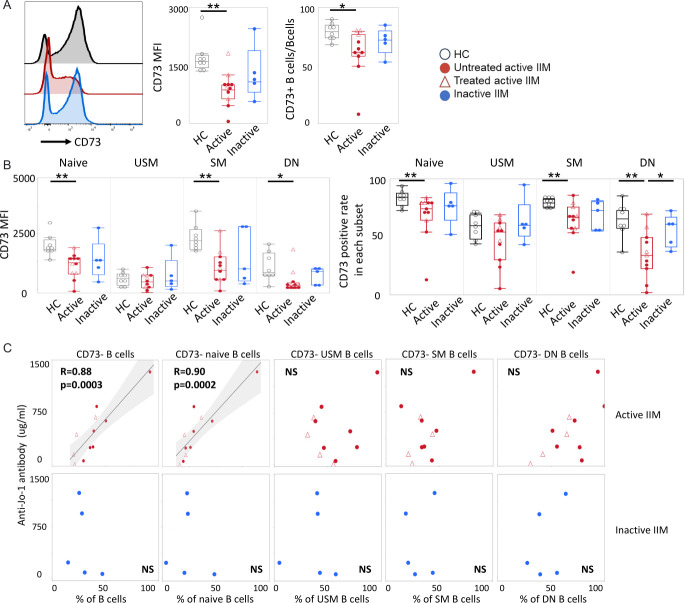
Low CD73 expression on B cells in active IIM. Results from manual gating of multiparameter flow cytometry analysis of B cells. (**A**) CD73 expression on total CD19+B cells in active IIM (n=11), inactive IIM (n=5) and HC (n=16) groups. (**B**) Comparison of expression levels of CD73 on naïve, USM, SM and DN B cells between the three groups. The median±IQR is shown. *p<0.05, **p<0.01 by Mann-Whitney U test. (**C**) Spearman’s correlation analysis between anti-Jo-1 antibody levels and proportion of CD73 negative B-cell subsets in active IIM (upper) and in inactive IIM (lower). The active IIM, inactive IIM and HC groups are coloured red, blue and black, respectively. DN, double-negative; HC, healthy controls; IIM, idiopathic inflammatory myopathies; SM, switched memory; USM, unswitched memory.

We next investigated B-cell activation by surface marker expression. We observed that CD95 expression was significantly higher on IgD+CD27+ USM B cells and IgD− CD27+SM B cells in both active IIM and inactive IIM compared with controls. However, the expression levels of HLA-DR, CD80 or CD11c on B-cell subsets were not different among the three groups (online supplemental figure 2). Intriguingly, while the expression level of CD39 on B cells was not different among these three groups (online supplemental figure 3A, B), CD73 expression was significantly lower on B cells in the active IIM group, which was confirmed by both the frequency and mean fluorescent intensity of expressing levels (figure 3A, online supplemental figure 3A). In order to clarify whether the low CD73 expression on B cells came from a shift in B-cell subpopulation composition, we examined the expression level of CD73 on each B-cell subpopulation. In the healthy control group, CD73 was expressed mainly on naïve B cells and SM B cells (figure 3B), which is consistent with a previous study.^23^ In the active IIM group, CD73 was significantly lower expressed on IgD+CD27− naïve B cells, IgD− CD27− DN B cells and IgD− CD27+SM B cells as compared with HC (figure 3B), which indicates that the expression of CD73 itself was downregulated in active IIM. Importantly, there was a positive correlation between the frequency of several CD73 negative B cell subsets and anti-Jo-1 antibody titre in the active IIM group, but not in the inactive IIM group (figure 3C). However, when we investigated the correlation between plasmablasts and autoantibody level, no correlation was found between the proportion of plasmablasts and anti-Jo-1 antibody levels (online supplemental figure 4). We further examined if the expression levels of CD73 on B cells may be influenced by methotrexate (MTX) usage. The CD73 expression on B cells was significantly higher in the patients treated with MTX (n=3) than in active patients with IIM who had not been treated with MTX (n=8, online supplemental figure 5).

### Activated B cells were expanded in active IIM

**Figure 4:**
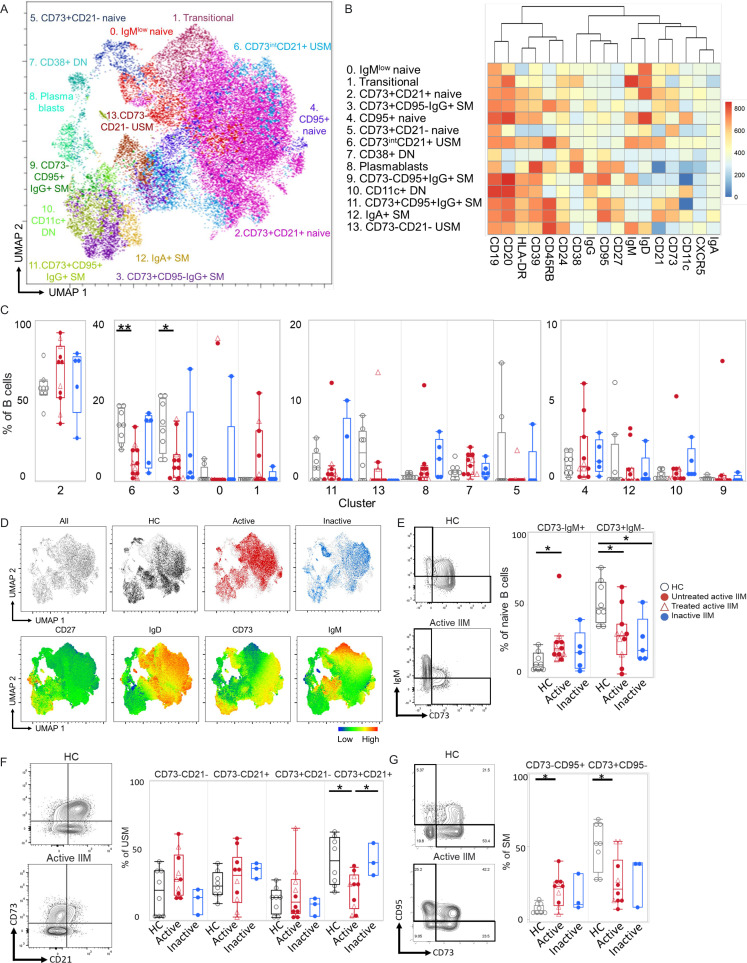
Expansion of activated B cells in active IIM. (**A**) UMAP visualisation of multiparameter flow cytometry analysis of B cells from active IIM group (n=11), inactive IIM group (n=5) and HC (n=8). (**B**) Heatmap of the expression levels of each flow cytometry marker in 14 clusters. (**C**) Comparison of the distribution of 14 clusters from FlowSOM in active IIM, inactive IIM and HC by Kruskal-Wallis test. (**D**) UMAP visualisation of B cells from all samples (grey), HC (black), active IIM (red) and inactive IIM (blue). (**E**) Manual gating for CD73− IgM+ and CD73+ IgM− naïve B cells in HC and active IIM, and the comparison among active IIM, inactive IIM and HC. (**F–G**) Representative gating for CD73− CD21− USM B cells and CD73− CD95+SM B cells, and the comparison among the three groups. The HC, untreated active, treated active and inactive IIM groups are indicated by black circles, red circles, red triangles and blue circles, respectively. The median±IQR is shown. *p<0.05, **p<0.01. HC, healthy controls; IIM, idiopathic inflammatory myopathies; SM, switched memory: UMAP, Uniform Manifold Approximation and Projection; USM, unswitched memory.

In order to further investigate which subpopulations differ between active IIM, inactive IIM and controls, we used unsupervised clustering visualised using UMAP and FlowSOM analyses to the pre-gated CD19+B cells from patients with active IIM (n=11), inactive IIM (n=5) and controls (n=8) (figure 4A,B). Notably, the cluster numbers were optimised by sequential validity testing to confirm that 14 clusters were biologically relevant for the data set (online supplemental methods and table 3). Comparing the distribution of the 14 clusters between patients with IIM and controls, we found that cluster 6 and cluster 3 had a significantly lower proportion in active IIM than in controls (figure 4C). The heatmap showed that cluster 6 included IgD+CD27+ USM B cells expressing CD21 and CD73, and cluster 3 included CD73+CD95− IgD− CD27+SM B cells (figure 4B). Moreover, a population of CD73− transitional B cells in cluster 1 was primarily detected in the active IIM group and neither in controls nor inactive IIM, and there was a trend for an increase in cluster 4 which reflected CD73 low naïve B cells (figure 4D). To validate the findings and further investigate CD73 together with other activation markers, we manually gated CD73− IgM+mature naïve B cells, CD73− CD21− USM B cells and CD73− CD95+SM B cells and compared these proportions with their CD73+/CD95− counterparts. CD73− IgM+naïve B cells and CD73− CD95+SM B cells, identifying activated B cells from different compartments, were significantly increased in active IIM compared with controls (figure 4E–G). On the other hand, CD73+CD21+ USM B cells were significantly lower in active IIM than in controls and inactive IIM, whereas CD73− CD21− USM B cells were not significantly different among the three groups (figure 4F). In summary, we detected a CD73 low activated B-cell phenotype in both naïve, USM and SM B cells in patients with active IIM, which can be speculated to reflect a hyperactivated state.

### Cytokine profiles in patients with IIM

**Figure 5:**
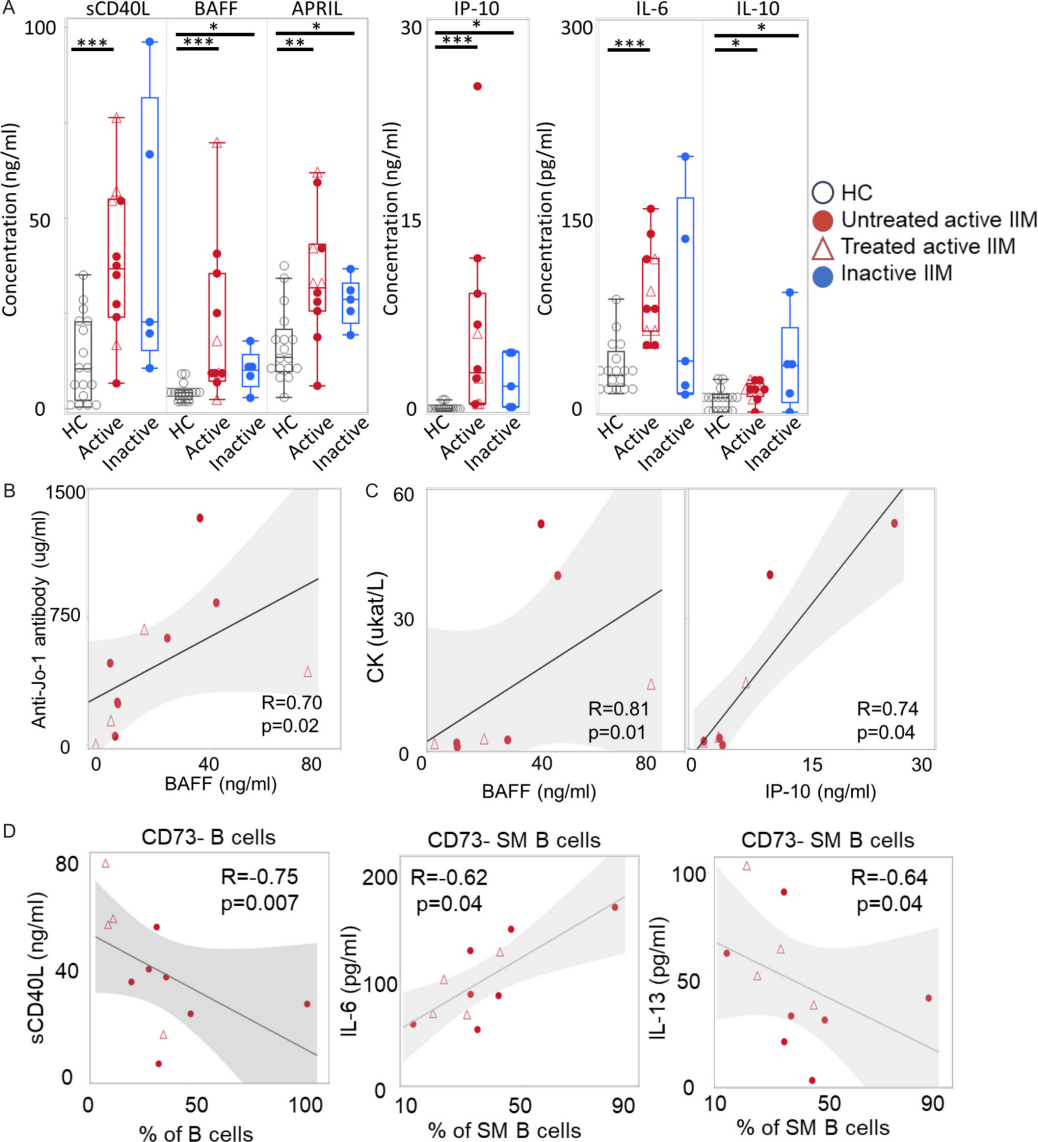
Evaluated proinflammatory cytokines in active IIM. (**A**) Comparison of serum cytokine levels between active IIM (n=11), inactive IIM (n=5) and HC (n=17). The median±IQR is shown. *p<0.05, **p<0.01, ***p<0.001 by Mann-Whitney U test. (**B**) Correlation between BAFF and anti-Jo-1 antibody levels by Spearman’s test. (**C**) Correlation between CK levels and BAFF and IP-10 by Spearman’s test. (**D**) Spearman’s correlation between selected cytokine levels and CD73− B cell population. The HC, untreated active, treated active and inactive IIM groups are indicated by black circles, red circles, red triangles and blue circles, respectively. APRIL, a proliferation-inducing ligand; BAFF, B-cell activating factor; CK, creatine kinase; HC, healthy controls; IIM, idiopathic inflammatory myopathies; IL, interleukin; IP-10, inducible protein-10; sCD40L, soluble CD40 ligand.

Lastly, we performed serum cytokine profiling to investigate if there was any association with B cell immunophenotypes. We measured 21 different cytokines in serum from 11 patients with active IIM and from 5 patients with inactive IIM and 17 healthy donors (online supplemental table 4). There were significant differences in the levels of sCD40L, BAFF, APRIL, IP-10, IL-6 and IL-10 between active IIM and HC, and in the levels of BAFF, APRIL, IP-10 and IL-10 between the inactive IIM group and controls (figure 5A). The level of BAFF correlated positively with anti-Jo-1 antibody levels and serum levels of CK. Similarly, the level of IP-10 correlated positively with CK levels (figure 5B,C). Next, we examined the correlation between CD73− B-cell subpopulations and the levels of cytokines in the patients with active IIM (online supplemental figure 6). Interestingly, CD73 negative B cells had a significant inverse correlation with sCD40L in active IIM (R=−0.75, p=0.007). In further analysis, the proportion of CD73− SM B cells correlated directly with IL-6 and inversely with IL-13 in active IIM (figure 5D). These trends were not significant when adjusting for multiple testing by the Benjamini-Hochberg procedure. In summary, the patients with active IIM displayed elevated levels of certain cytokines and inflammation markers which reflect immune dysregulation that could be mediating B-cell activation.

## Discussion

Here we report detailed immunophenotyping data of peripheral B cells and cytokine profiles in serum from patients with anti-Jo-1 antibody positive IIM. These results revealed significant changes in the B cells of patients with anti-Jo-1 antibody positive IIM, in particular a low CD73 expression on B cells in patients with inflammatory active disease. Importantly, CD73 negative B cells correlated with anti-Jo-1 antibody levels, disease activity as indicated by serum levels of CK and with levels of proinflammatory cytokines such as BAFF and IP-10.

Several studies have reported changes in major B-cell subpopulations in anti-Jo-1 antibody positive IIM.^31^,^33^ Consistent with previous studies, we demonstrate that peripheral memory B cells were decreased and transitional B cells increased in patients with untreated active IIM compared with HC. In certain subgroups of IIM, an expansion of plasmablasts has previously been observed,^19^ but in our study, the frequency of plasmablasts was not different between patients with IIM and controls. However, detection might be influenced by cryopreservation, which can reduce the number of viable plasmablasts in the samples.^34^ Notably, B-cell distortions may be affected by treatment, and a previous study showed that immunosuppressive therapy in anti-Jo-1 antibody positive IIM can result in a normalisation of transitional B cells.^18^ B cells can also be found in tissue with evidence of local activation and expansion^31^,^33^ and it has been hypothesised that the altered naïve and memory B cell balance may be explained by cells infiltrating into inflammatory sites such as muscles or lungs. Hence, combining previous reports with our data may indicate that the bone marrow output of B cells could be increased during high disease activity and that memory B cells have migrated into affected tissues. In line with an increased production of B cells we could detect an expansion of circulating transitional B cells in some of the patients with active IIM. Notably, transitional B cells display low levels of CD73 expression which increase as they mature into follicular B cells.^35^ However, an elevated subpopulation of CD73− IgM+IgD+ CD27− mature naïve B cells was still found after excluding transitional B cells, reflecting increased activation of naïve B cells. For SM and USM populations, CD73 was investigated together with other activation markers. The Fas-receptor CD95 is expressed following B-cell activation,^21 22^ while CD21, a co-receptor for the BCR, is downregulated after B-cell activation.^36^ We could observe expansions of CD73− CD95+SM B cells and CD73− CD21− USM B-cell subpopulations with an activated phenotype in patients with active IIM. Overall, active IIM showed expansion of B cells with low CD73 expression across different B cell compartments including naïve, USM and SM B cells. Moreover, low CD73 B cells have been described to be expanded in several diseases of chronic infectious diseases such as HIV and hepatitis C virus in addition to autoimmune diseases such as juvenile idiopathic arthritis or systemic lupus erythematosus.^37^,^40^ It is possible that chronic stimulation of immune cells might lead to a reduction of CD73 expression. Interestingly, CD73 contributes to the ATP pathway, where CD39 converts ATP into AMP and CD73 subsequently dephosphorylates AMP into adenosine.^25^ ATP has proinflammatory effects via receptors such as P2X7 and mediates the release of proinflammatory cytokines.^41^ On the other hand, adenosine can have anti-inflammatory functions by inhibition of lymphocyte proliferation or regulation of proinflammatory cytokines.^26 27^ Therefore, a lack of CD73 on B cells may inhibit the metabolism of AMP to adenosine, resulting in maintained low adenosine concentrations. In our study, we identified changes in CD73 B cell expression in patients with IIM, pointing towards alteration of the ATP/adenosine pathway, potentially leading to B-cell hyperactivation in anti-Jo-1 antibody positive IIM. On the other hand, CD39 expression levels on B cells in IIM did not differ between anti-Jo-1 antibody positive IIM and HC. While the anti-inflammatory function of CD39 on regulatory T cells is well known, and low CD39 expression on regulatory T cells has been reported in rheumatoid arthritis,^25^ the expression patterns may depend on cell types and autoimmune diseases.

Interestingly, the ATP/adenosine pathway can be a therapeutic target in autoimmune rheumatic diseases. For instance, MTX increases adenosine levels in rheumatoid arthritis.^42^ MTX inhibits 5-aminoimidazole-4-carboxamide ribonucleotide (AICAR) transformylase, leading to increased AICAR which blocks adenosine deaminase and AMP deaminase.^43 44^ Hence, MTX effectiveness can be explained by the adenosine signalling pathway. A previous study showed that in anti-aminoacyl t-RNA synthetases antibodies positive IIM, differential gene expression analysis on blood-derived plasmablasts from active patients and from inactive patients showed increased ATP synthesis-related genes.^20^ In our study, MTX-treated patients showed high CD73 expression on B cells, which might indicate that MTX could influence CD73 expression also when the disease activity was high. However, longitudinal studies before and after treatment with a larger number of patients and adjusting for glucocorticoid use are warranted.

Moreover, we here demonstrate striking differences in cytokine profiles in patients with IIM compared with controls, which correlated with changes in B-cell phenotype. Consistently, in other cytokine studies of anti-Jo-1 antibody positive IIM, the level of BAFF in peripheral blood was reported to be high and correlate with anti-Jo-1 antibody levels and disease activity in anti-Jo-1 antibody positive IIM.^45^,^47^ Moreover, IL-6, IL-8, IL-10, IP-10 and TNF-α have been reported to be elevated in serum of patients with IIM.^48^,^50^ Interestingly, ligation of BAFF to BAFF-receptor leads to increased ATP^51^ and the levels of IP-10 and IL-8 were decreased by inhibition of ATP signalling in rats.^52^ In our study, the levels of BAFF and IP-10 were still high in inactive IIM, whereas sCD40L and IL-6 were decreased as the disease activity stabilised, suggesting that the cytokine profiles could change with disease activity. In IIM, activation of B cells and migration of immune cells may be maintained through the disease course via the high levels of BAFF and IP-10, which were associated with CD73− DN B cell subpopulations (online supplemental figure 6). We were also able to reveal an association between CD73− SM B-cell subpopulations with IL-6 and IL-8 levels, which previously have been reported to be released by ATP ligation.^53 54^ The negative correlation between sCD40L and the proportion of CD73− B cells and between IL-13 and CD73− SM B cells can be hypothesised to reflect feedback function; however, further studies are required for drawing conclusions. Altogether, this may indicate a direct link between the skewed CD73− B-cell subpopulations, the ATP/adenosine pathway and the inflammatory cytokine profiles in anti-Jo-1 antibody positive IIM. Moreover, our results support that CD73− B-cell subpopulations may be involved in the pathogenesis of anti-Jo-1 antibody positive IIM via ATP/adenosine pathway.

There are some limitations in this study. First, the total number of patients analysed in the study was small, since IIMs are rare diseases with prevalence from 2 to 25 per 100 000 people.^55^ However, this is a unique cohort with well-characterised clinical history and focused on patients being positive for IgG anti-Jo-1 antibodies and with differences in disease activity. Hence, although our results were significant, the small cohort size merits further validation with more patients and other methodology. Second, it was not within the scope of this study to fully delineate the function of the CD73− B-cell subpopulations. Notably, our results indirectly showed that CD73− B cells may be involved in the pathogenesis of anti-Jo-1 antibody positive IIM and future functional assays are merited.

In conclusion, we report a significantly lower expression of CD73 on peripheral B cells from patients with anti-Jo-1 antibody positive IIM compared with HC. This may lead to hyperactivation of B cells through the ATP/adenosine pathway and may thus have a role in autoantibody production and in the pathogenesis of anti-Jo-1 antibody positive IIM.

## Supplementary material

10.1136/rmdopen-2024-005401 online supplemental file 1

## Data Availability

Data are available upon reasonable request.
