# Supplementary material for: CD73low B-cell phenotypes and distinct cytokine profiles in patients with active anti-Jo-1 antibody positive idiopathic inflammatory myopathies
Source: RMD Open. 2025 Apr 9;11(2):e005401. doi: 10.1136/rmdopen-2024-005401 (PMC11987157; doi:10.1136/rmdopen-2024-005401)
Supplement: online supplemental file 1 [file rmdopen-11-2-s001.docx]

Supplementary Information

**CD73^low^ B cell phenotype and distinct cytokine profiles in active anti-Jo-1 antibody positive idiopathic inflammatory myopathies**

Contents

Supplementary methods. Optimizing the number of clusters in FlowSOM analysis

Supplementary figure 1. The comparison of transitional B cells

Supplementary figure 2. Expression of activation marker on B cell subsets

Supplementary figure 3. Gating plot of CD73+ B cells and CD39+ B cells and expression of CD39 on B cells

Supplementary figure 4. Correlation between the proportion of plasmablasts and anti-Jo-1 antibody titer

Supplementary figure 5. CD73 expression on B cells between patients treated with MTX and active IIM

Supplementary figure 6. Correlation analysis between B cell subsets and the leevels of cytokines

Supplementary table 1. Flow cytometry panel

Supplementary table 2. Frequency of B cells

Supplementary table 3. Clustering analysis by FlowSOM

Supplementary table 4. The levels of 21 kinds of cytokines

**Supplementary methods**

**Optimizing the number of clusters in FlowSOM analysis**

First, we addressed how to decide the number of the clusters. We examined the characteristics of each cluster for each different number of clusters and examined the validity (Supplementary table 2). For instance, the level of CD21 expression was intermediate in cluster 6 in 13 clusters, however, it was separated into two clusters (cluster 6 and 13) in 14 clusters by the expression levels of CD21 which was reasonable. On the other hand, compared 14 clusters to 15 clusters, IgG+ USM cluster (cluster 11) was separated into 2 clusters in 15 clusters; cluster 12 (1.74%) and cluster 11 (0.42%) which was a too small population. Therefore, we decided the number of clusters should be 14.


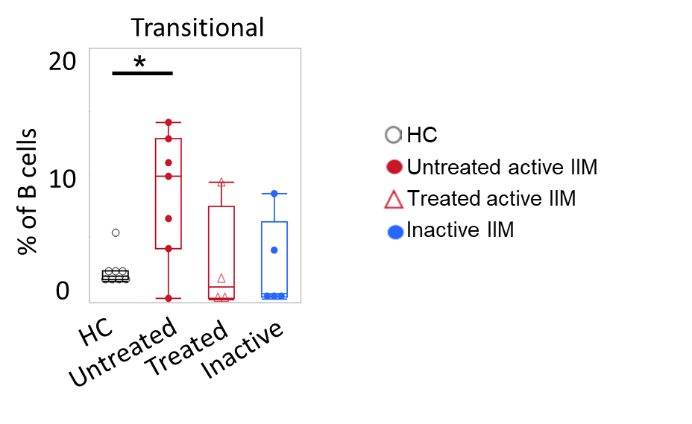


**Supplementary figure 1. The comparison of transitional B cells**

Flow cytometry results showing the frequency of CD19+ CD24^hi^ CD38+ transitional B cells among CD19+ B cells in healthy controls (HC, n = 8), the untreated active IIM (n = 7), treated active IIM (n = 4) and inactive IIM groups (n = 5) indicated by black circles, red circles, red triangles and blue circles,, respectively. The median ± interquartile range is shown. *P<0.05 by Mann-Whitney U test.

**
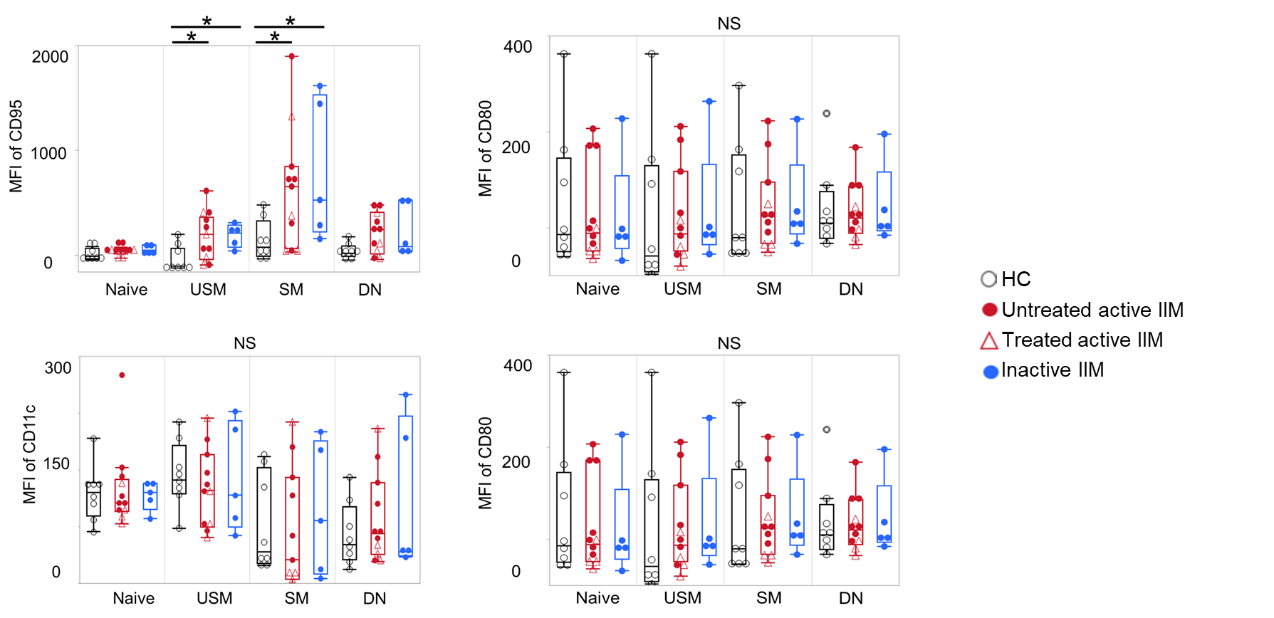
**

**Supplementary figure 2. Expression of activation marker on B cell subsets**

Flow cytometry results showing MFI of indicated surface markers in naïve B cells, unswitched memory B cells (USM), switched memory (SM) B cells and double negative (DN) cells in healthy controls (HC), active IIM and inactive IIM groups, indicated by black, red and blue marker, respectively. The median ± interquartile range is shown. *P<0.05 by Mann-Whitney U test.

**
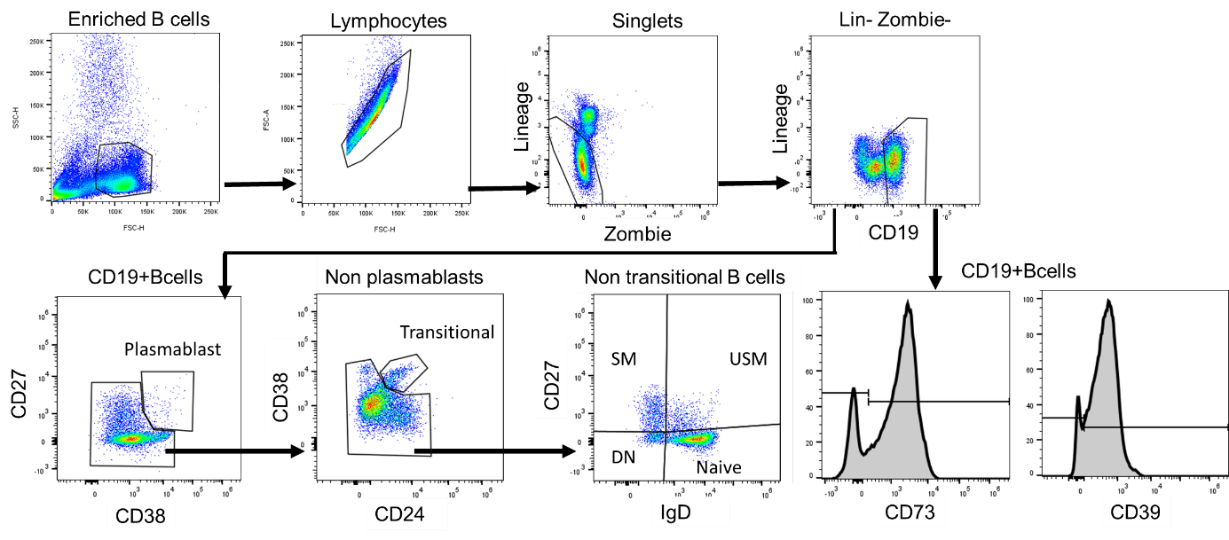
**A

**
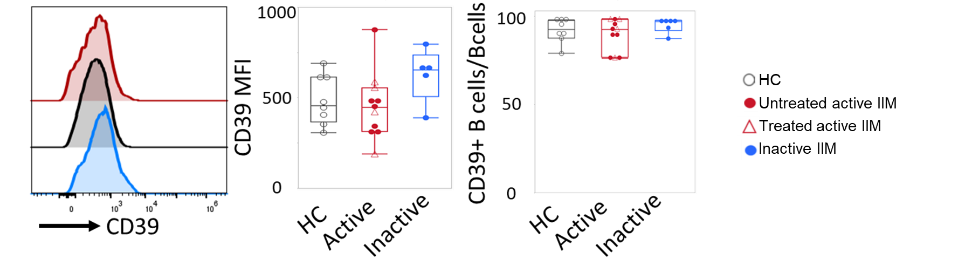
**

B

**Supplementary figure 3. Gating plot of CD73+ B cells and CD39+ B cells and expression of CD39 on B cells**

A, Gating plot of CD73+ B cells and CD39+ B cells. B, Flow cytometry results showing MFI of CD39 in all CD19+ B cells in HC, active IIM and inactive IIM groups, as indicated by black, red and blue marker, respectively. The median ± interquartile range is shown.


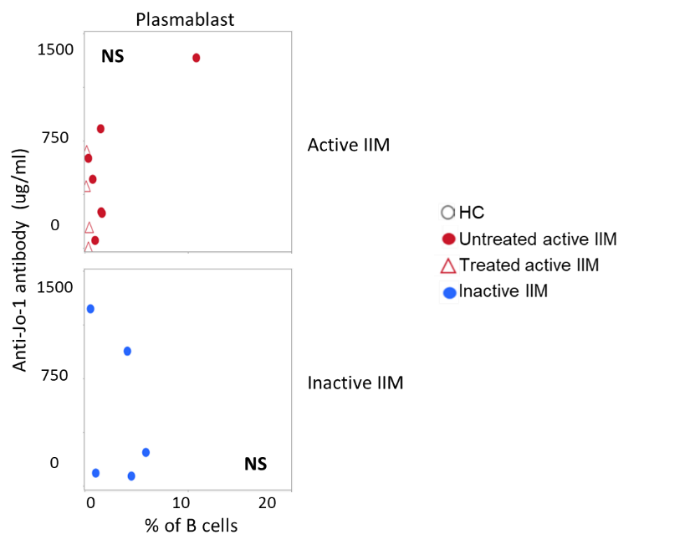


**Supplementary figure 4. Correlation between the proportion of plasmablasts and anti-Jo-1 antibody titer**

Correlation by serum levels of IgG anti-Jo1 by ELISA and frequencies of plasma blasts among CD19+ B cells. The HC, untreated active, treated active and inactive IIM groups are indicated by black circles, red circles, red triangles and blue circles, respectively. by Spearman's correlation coefficient was used for this analysis.


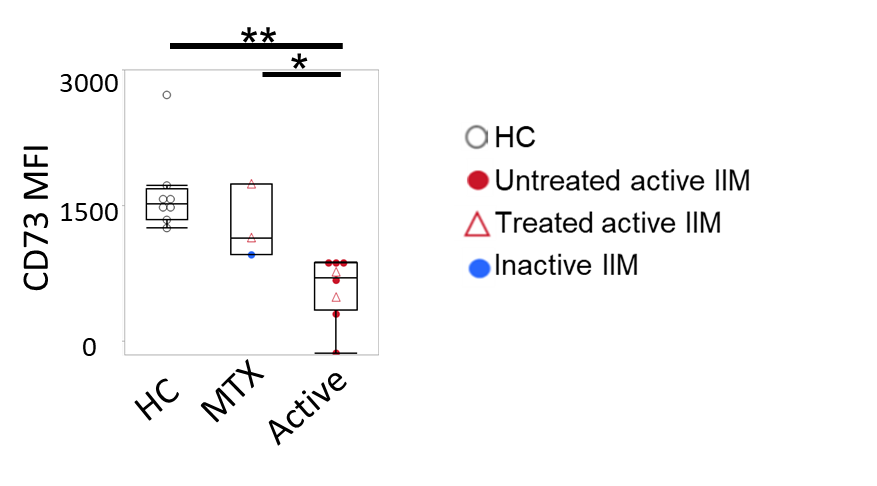


**Supplementary figure 5. CD73 expression on B cells between patients treated with MTX and active IIM**

The HC, untreated active, treated active and inactive IIM groups are indicated by black circles, red circles, red triangles and blue circles, respectively. The median ± interquartile range is shown. *P<0.05, **P<0.01 by Mann-Whitney U test.


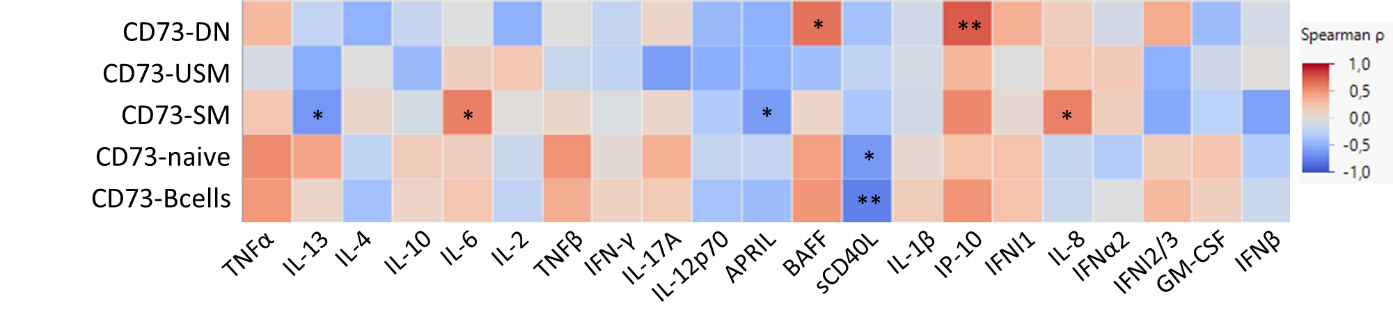


**Supplementary figure 6. Correlation analysis between B cell subsets and the levels of cytokines**

The heatmap of 21 cytokines and B cell subpopulations in active IIM visualizing the correlation coefficients by Spearman’s test in active. *P<0.05, **P<0.01 without adjustment for multiple testing.

**Supplementary table 1. Flow cytometry panel**

| Fluorochrome | Antigen | Clone | Company |
| --- | --- | --- | --- |
| Brilliant Violet 421 | CD73 | AD2 | BD |
| Pacific Blue | CD20 | 2H7 | BioLegend |
| Brilliant Violet 480 | CD38 | HIT2 | BD |
| Brilliant Violet 510 | CD19 | SJ23C1 | BD |
| Brilliant Violet 570 | IgM | MHM-88 | BioLegend |
| Brilliant Violet 605 | IgD | AIA6-2 | BioLegend |
| Brilliant Violet 650 | HLA-DR | G46-6 | BD |
| Brilliant Violet 711 | CD22 | HIB22 | BD |
| Brilliant Violet 750 | CXCR5 | J252D4 | BioLegend |
| Brilliant Violet 785 | CD80 | 2D10 | BioLegend |
| BB515 | CD24 | ML5 | BD |
| BB700 | CD27 | O323 | BD |
| PerCP-eFlour710 | CD39 | eBioA1 (A1) | invitrogen |
| PE-Cy7 | CD11c | 3.9 | BioLegend |
| APC | CD95 | DX2 | BioLegend |
| AF647 | CD45RB | MEM-55 | invitrogen |
| AF700 | CD21 | Bu32 | BioLegend |
| APC-H7 | CD3 | SK7 | BD |
| APC-H7 | CD14 | MφP9 | BD |
| APC-H7 | CD16 | 3G8 | BD |
| Zombie NIR | Fixable Dye |  | BioLegend |
| Alexa Fluor 532 | IgG* | G18-145 | BD |
| NovaFluor Blue 610 | IgA** | G18-1 | BD |

*IgG was conjugated with AF532 using Alexa Fluor™ 532 Antibody Labeling Kit (Invitrogen).

**IgA was conjugated with NovaFluor Blue 610/70S using NovaFluor™ Blue 610/70S Conjugation Kit (Invitrogen).

**Supplementary table 2. Frequency of B cells**

|  | HC (n =8) | Active IIM (n =11) | Inactive IIM (n =5) | P value |
| --- | --- | --- | --- | --- |
| PBMCs after thawing (x106) | 25 (14-30) | 9 (5-10) | 5 (4-13) |  |
| B cells after isolation (x 105) | 20 (10-23) | 7 (4-10) | 6 (4-9) |  |
| Frequency of B cells in PBMCs (%) | 9 (6-11) | 9 (8-10) | 10 (6-15) | NS |

The median ± interquartile range is shown. NS; not significant by Mann-Whitney U test.

**Supplementary table 3. Clustering analysis by FlowSOM**

| 15 clusters | % |  | 14 clusters | % |  | 13 clusters | % |  | 12 clusters | % |  | 11 clusters | % |
| --- | --- | --- | --- | --- | --- | --- | --- | --- | --- | --- | --- | --- | --- |
| 1. Transitional | 2.92 | **-** | 1. Transitional | 2.92 | - | 1. Transitional | 2.92 | **-** | 1. Transitional | 2.92 | **-** | 1. Transitional | 2.92 |
| 0. IgM^low^ naive | 4.88 | **-** | 0. IgM^low^ naive | 4.88 | - | 0. IgM^low^ naive | 4.88 | > | 0. CD21int naïve | 6.38 | **-** | 0. CD21int naïve | 6.38 |
| 5. CD73+ CD21- naive | 1.50 | **-** | 5. CD73+ CD21- naive | 1.50 | - | 5. CD73+ CD21- naive | 1.50 |  |  |  |  |  |  |
| 6. CD73^int^ CD21+ USM | 9.14 | **-** | 6. CD73^int^ CD21+ USM | 9.14 | > | 6. CD73^int^ CD21intUSM | 11.1 | **-** | 11. CD95-CD21+CD11c-USM | 8.12 | **-** | 10. CD95-CD21+CD11c-USM | 8.12 |
| 14. CD73- CD21- USM | 1.94 | **-** | 13. CD73- CD21- USM | 1.94 |  |  |  | > | 2. CD21+ naive | 65.1 | > | 2. CD21+ naive | 66.5 |
| 2. CD73+ CD21+ naive | 62.2 | **-** | 2. CD73+ CD21+ naive | 62.2 | - | 2. CD73+ CD21+ naive | 62.2 |  |  |  |  |  |  |
| 4. CD95+ naive | 1.43 | **-** | 4. CD95+ naive | 1.43 | - | 4. CD95+ naive | 1.43 | **-** | 4. CD95+ naive | 1.43 |  |  |  |
| 3. CD73+CD95-IgG+ SM | 8.77 | **-** | 3. CD73+CD95-IgG+ SM | 8.77 | - | 3. CD73+CD95-IgG+ SM | 8.77 | **-** | 3. CD73+CD95-IgG+ SM | 8.77 | **-** | 3. CD73+CD95-IgG+ SM | 8.77 |
| 7. CD38+ DN | 1.52 | **-** | 7. CD38+ DN | 1.52 | - | 7. CD38+ DN | 1.52 | **-** | 5. CD38+ DN | 1.52 | **-** | 4. CD38+ DN | 1.52 |
| 8. Plasmablast | 1.64 | **-** | 8. Plasmablast | 1.64 | - | 8. Plasmablast | 1.64 | **-** | 6. Plasmablast | 1.64 | **-** | 5. Plasmablast | 1.64 |
| 9. CD73- CD95+ IgG+ SM | 0.43 | **-** | 9. CD73- CD95+ IgG+ SM | 0.43 | - | 9. CD73- CD95+ IgG+ SM | 0.43 | **-** | 7. CD73- CD95+ IgG+ SM | 0.43 | **-** | 6. CD73- CD95+ IgG+ SM | 0.43 |
| 10. CD11c+ DN | 0.68 | **-** | 10. CD11c+ DN | 0.68 | - | 10. CD11c+ DN | 0.68 | **-** | 8. CD11c+ DN | 0.68 | **-** | 7. CD11c+ DN | 0.68 |
| 11. CD95+ IgG+USM | 0.42 | > | 11. CD73+ CD95+ IgG+ USM | 2.16 | - | 11. CD73+ CD95+ IgG+ USM | 2.16 | **-** | 9. CD73+ CD95+ IgG+ USM | 2.16 | **-** | 8. CD73+ CD95+ IgG+ USM | 2.16 |
| 12. CD73intIgG+SM | 1.74 |  |  |  |  |  |  |  |  |  |  |  |  |
| 13. IgA+ SM | 0.85 | **-** | 12. IgA+ SM | 0.85 | - | 12. IgA+ SM | 0.85 | **-** | 10. IgA+ SM | 0.85 | **-** | 9. IgA+ SM | 0.85 |

**Supplementary table 4. The levels of 21 kinds of cytokines**

|  | HC (n =17) | Active IIM (n =11) | Inactive IIM (n =5) |
| --- | --- | --- | --- |
| TNFa (pg/ml) | 94.8 (59.3 - 167.6) | 99.8 (47.4 - 170.6) | 108.1 (0 - 452.7) |
| IL-13 (pg/ml) | 31.2 (20.4 - 34.5) | 37.6 (27.6 - 59.9) | 37.4 (10.0 - 87.4) |
| IL-4 (pg/ml) | 138.3 (94.4 - 434.1) | 300.6 (227.9 - 440.0) | 506.3 (94.6 - 1210.0) |
| IL-10 (pg/ml) | 10.8 (0 - 13.5) | 17.3 (12.2 - 21.9) * | 35.6 (7.2 - 64.7) * |
| IL-6 (pg/ml) | 28.0 (19.7 - 46.4) | 79.6 (61.7 - 117.7) *** | 39.0 (14.6 - 164.0) |
| IL-2 (pg/ml) | 469.8 (351.0 - 497.0) | 319.5 (0 - 392.0) | 501.9 (138.2 - 1109.2) |
| TNFb (pg/ml) | 210.9 (93.4 - 467.8) | 181.0 (0 - 454.6) | 74.7 (16.2 - 853.3) |
| IFNg (pg/ml) | 1739.3 (943.7 - 2503.5) | 1999.6 (575.0 - 6236.3) | 1601.8 (624.4 - 6657.4) |
| IL-17A (pg/ml) | 12.6 (8.0 - 22.5) | 11.6 (9.0 - 34.6) | 5.9 (2.3 - 17.1) |
| IL-12p70 (pg/ml) | 36.7 (24.6 - 46.3) | 74.4 (16.4 - 132.2) | 75.1 (8.5 - 122.3) |
| APRIL (ng/ml) | 13.4 (9.7 - 20.8) | 31.7 (25.6 - 43.2)** | 28.6 (22.4 - 32.8) * |
| BAFF (ng/ml) | 3.2 (2.4 - 4.2) | 9.4 (7.1 - 35.4)*** | 10.0 (5.7 - 14.2) * |
| sCD40L (ng/ml) | 10.4 (2.3 - 22.7) | 36.7 (24.0 - 54.9)*** | 22.7 (15.2 - 81.4) |
| IL-1b (pg/ml) | 51.6 (0 - 97.4) | 54.7 (0 - 82.8) | 0 (0 - 98.7) |
| IP-10 (ng/ml) | 0.3 (0.1 - 0.5) | 3.0 (0.7 - 9.1)*** | 2.0 (0.4 - 4.5)* |
| IFNl1 (pg/ml) | 351.8 (0 -727.1) | 355.3 (0 - 768.4) | 305.1 (0 - 776.2) |
| IL-8 (pg/ml) | 22.5 (17.7 - 41.3) | 16.7 (15.6 - 23.0) | 12.1 (7.4 - 69.4) |
| IFNa2 (pg/ml) | 4.5 (1.1 - 14.7) | 3.3 (1.2 - 8.6) | 5.4 (1.1- 36.6) |
| IFNl2/3 (pg/ml) | 596.2 (217.6 - 848.2) | 987.0 (594.2 - 1145.3) | 652.3 (178.7 - 896.7) |
| GM-CSF (pg/ml) | 7.3 (2.0 - 10.4) | 4.1 (0 - 7.8) | 11.6 (0 - 16.1) |
| IFNb (pg/ml) | 75.0 (0 - 110.3) | 0 (0 - 0) | 0 (0 - 810.2) |

The median ± interquartile range is shown. *P<0.05, **P<0.01, ***P< 0.001 compared to HC by Mann-Whitney U test.
